# Individual risk factors predictive of major trauma in pre-hospital injured older patients: a systematic review

**DOI:** 10.29045/14784726.2022.03.6.4.26

**Published:** 2022-03-01

**Authors:** Abdullah Pandor, Gordon Fuller, Munira Essat, Lisa Sabir, Chris Holt, Helen Buckley Woods, Hridesh Chatha

**Affiliations:** The University of Sheffield ORCID iD: https://orcid.org/0000-0003-2552-5260; The University of Sheffield ORCID iD: https://orcid.org/0000-0001-8532-3500; The University of Sheffield ORCID iD: https://orcid.org/0000-0003-2397-402X; The University of Sheffield ORCID iD: https://orcid.org/0000-0001-6488-3314; The University of Sheffield; The University of Sheffield; The University of Sheffield

**Keywords:** emergency medical services, triage, wounds and injuries

## Abstract

**Background:**

Older adults with major trauma are frequently under-triaged, increasing the risk of preventable morbidity and mortality. The aim of this systematic review was to identify which individual risk factors and predictors are likely to increase the risk of major trauma in elderly patients presenting to emergency medical services (EMS) following injury, to inform future elderly triage tool development.

**Methods:**

Several electronic databases (including Medline, EMBASE, CINAHL and the Cochrane Library) were searched from inception to February 2021. Prospective or retrospective diagnostic studies were eligible if they examined a prognostic factor (often termed predictor or risk factor) for, or diagnostic test to identify, major trauma. Selection of studies, data extraction and risk of bias assessments using the Quality in Prognostic Studies (QUIPS) tool were undertaken independently by at least two reviewers. Narrative synthesis was used to summarise the findings.

**Results:**

Nine studies, all performed in US trauma networks, met review inclusion criteria. Vital signs (Glasgow Coma Scale (GCS) score, systolic blood pressure, respiratory rate and shock index with specific elderly cut-off points), EMS provider judgement, comorbidities and certain crash scene variables (other occupants injured, occupant not independently mobile and head-on collision) were identified as significant pre-hospital variables associated with major trauma in the elderly in multi-variable analyses. Heart rate and anticoagulant were not significant predictors. Included studies were at moderate or high risk of bias, with applicability concerns secondary to selected study populations.

**Conclusions:**

Existing pre-hospital major trauma triage tools could be optimised for elderly patients by including elderly-specific physiology thresholds. Future work should focus on more relevant reference standards and further evaluation of novel elderly relevant triage tool variables and thresholds.

## Background

Major trauma, defined as life-threatening or life-changing significant injury, represents an important public health burden (National Institute for Health and Care Excellence [NICE] 2016; Thompson et al., 2021). Globally, there are over 5 million deaths each year and considerably more people are temporarily or permanently disabled (Haagsma et al., 2016; World Health Organization, 2005). Pre-hospital triage is a major component of the trauma care system and adequate identification of significant injuries with direct transportation of patients to a specialist major trauma centre (MTC) may improve survival and functional outcomes (Moran et al., 2018). Early pre-hospital trauma evaluation can be a challenge to emergency medical services (EMS) professionals, as the presence of major trauma at the scene is not always obvious and clinical assessment can be more difficult in challenging field situations (Newgard et al., 2018).

EMS professionals currently use major trauma triage tools to help them to recognise whether a patient is seriously injured or not. Pre-hospital trauma triage criteria often include a combination of physiological, anatomical and mechanism of injury parameters as predictors of severe injury (van Rein et al., 2017). While injuries in older adults are increasing (Coats & Lecky, 2017), current pre-hospital triage systems fail to identify a large proportion of elderly trauma patients with major trauma and are subject to under-triage, increased mortality and poorer outcomes (Banerjee et al., 2017). Older adults are a particularly vulnerable population and tend to have more cognitive and physical impairments and can incur serious injuries from low-energy trauma mechanisms (Newgard et al., 2014). In addition, pre-existing medical conditions, frailty and medications (e.g. anticoagulants, antiplatelet) can alter the response to injury, confound clinical evaluation and influence the initial EMS decision to convey to a destination facility (Cox et al., 2014).

Despite the publication of various pre-hospital major trauma triage protocols for the elderly (Fuller et al., 2021), the development, derivation and validation of these triage tools is often unclear. In addition, these models often employ and arbitrarily assign different sets of risk factors and predictors to identify major trauma without adequate supporting evidence. The aim of this systematic review is to identify which individual risk factors and predictors are likely to increase the risk of major trauma in elderly patients presenting to EMS following injury, to inform future elderly triage tool development.

## Methods

### Study design

The systematic review was undertaken in accordance with the general principles recommended in the Preferred Reporting Items for Systematic Reviews and Meta-Analyses (PRISMA) statement (Moher et al., 2009). This review was part of a larger project on major trauma triage, which was registered on the PROSPERO international prospective register of systematic reviews (CRD42020150342). The full protocol is available at https://fundingawards.nihr.ac.uk/award/17/16/04.

### Eligibility criteria

All prospective or retrospective studies were eligible if they included elderly adults with suspected serious injury evaluated by land EMS personnel before arrival to hospital; and evaluated any pre-hospital variable as a prognostic factor (often termed predictor or risk factor) or diagnostic test, for major trauma that would benefit from MTC care. Possible pre-hospital predictors could include any variable feasibly measured by EMS personnel – for example, demographic, anatomical location of injury, physical examination finding, vital sign value or mechanism of injury parameters. There is no accepted definition for major trauma that would benefit from MTC care. Eligible studies could therefore include any recognised reference standard for major trauma, including Injury Severity Score (ISS), resource-based measures (Lerner et al., 2014; Vassallo et al., 2020), mortality or a composite endpoint (Linn, 1995). The relationship between pre-hospital variables and major trauma benefitting from MTC care could be evaluated using measures of association (e.g. odds ratios) or diagnostic accuracy metrics (e.g. sensitivity, specificity).

While a standard consensus definition of elderly has not been established, a cut-off of over 60 years was used in this study. This is consistent with official definitions commonly used in studies of older adults (Shenkin et al., 2017) by the World Health Organization (2018), the United Nations (2019) and in previous major trauma triage research (Benjamin et al., 2018). Moreover, an inflection in frailty and mortality after trauma have been shown at approximately this age threshold (Hanlon et al., 2018; Kuhne et al., 2005). If studies included participants less than 60 years of age, we included the study if it was possible to identify the ratio of participants who were more than 60 years of age; if the ratio was more than 75% we included these studies. In case of studies with mixed populations – for example, data for adults above and below 60 years of age, which could not be separated – the study authors were contacted to provide the data separately for the group of interest, where possible.

Studies including people presenting to the emergency department via non-EMS – that is, private transportation or air transport – were excluded. Research investigating the overall performance of triage tools or protocols was also not eligible.

### Data sources and searches

A systematic literature search was conducted across nine electronic databases including Medline (1946 to February 2021), EMBASE (1974 to February 2021), CINAHL (1981 to February 2021) and the Cochrane Library (2021, issue 2). The search strategy used free text and thesaurus terms and combined synonyms relating to the condition (e.g. pre-hospital trauma triage) with risk factor assessment or risk prediction modelling terms. No language or date restrictions were used. Searches were supplemented by hand-searching the reference lists of all relevant studies, performing a citation search of relevant articles, contacting key experts in the field and undertaking systematic keyword searches of the World Wide Web using the Google search engine. Further details on the search strategy can be found in Supplementary 1.

### Study selection

All titles were examined for inclusion by one reviewer (ME) and any citations that clearly did not meet the inclusion criteria (e.g. non-human, unrelated to pre-hospital major trauma triage) were excluded. All abstracts and full-text articles were then examined independently by two reviewers (ME and HC or AP and LS). Any disagreements in the selection process were resolved through discussion or, if necessary, arbitration by a third reviewer (GF) and included by consensus.

### Data extraction and risk of bias assessment

Data relating to study design, methodological quality and outcomes were extracted by one reviewer (ME or AP) into a standardised data extraction form and independently checked for accuracy by a second (CH, HC or LS). Any discrepancies were resolved through discussion to achieve agreement. Where differences were unresolved, a third reviewer’s opinion was sought (GF). Where multiple publications of the same study were identified, data were extracted and reported as a single study. In the contingency that multiple adjusted analyses were reported, the multi-variable model including the most predictive variables was chosen.

The methodological quality of each included study was assessed using the Quality in Prognostic Studies (QUIPS) tool (Hayden et al., 2013). This instrument assesses the risk of bias in six domains: study participation, study attrition, prognostic factor measurement, outcome measurement, adjustment for other prognostic factors and statistical analysis and reporting. For this review, items on confounding were not considered relevant because in studies regarding prognosis, the aim is to predict a specific outcome (i.e. major trauma) based on individual or in combination with several other prognostic factors, rather than investigate causation (Kent et al., 2020). To guide the overall domain-level judgement about whether a study is at high, moderate or low risk of bias, sub-domains within each domain include a number of signalling questions to help judge risk of bias. An overall risk of bias for each individual study was defined as low risk when three or more domains were low risk and none were high risk; and high risk of bias when one or more domains were considered high risk. Studies were assigned a moderate risk of bias if three or more domains were moderate risk and none were high risk (Hayden et al., 2013).

### Data synthesis and analysis

Due to significant levels of heterogeneity between studies, variable reporting of items and the high risk of attributable bias, meta-analysis was not possible. Therefore, we analysed the association or prediction between prognostic factors (e.g. pre-hospital demographic, clinical or injury parameters) and a final diagnosis of major trauma using a narrative review approach (as recommended by the Cochrane Collaboration (McKenzie et al., 2019) and the Centre for Reviews and Dissemination (2009) for undertaking systematic reviews). All analyses were conducted using Microsoft Excel 2010 (Microsoft Corporation, Redmond, WA, USA).

## Results

### Study flow

**Figure 1:**
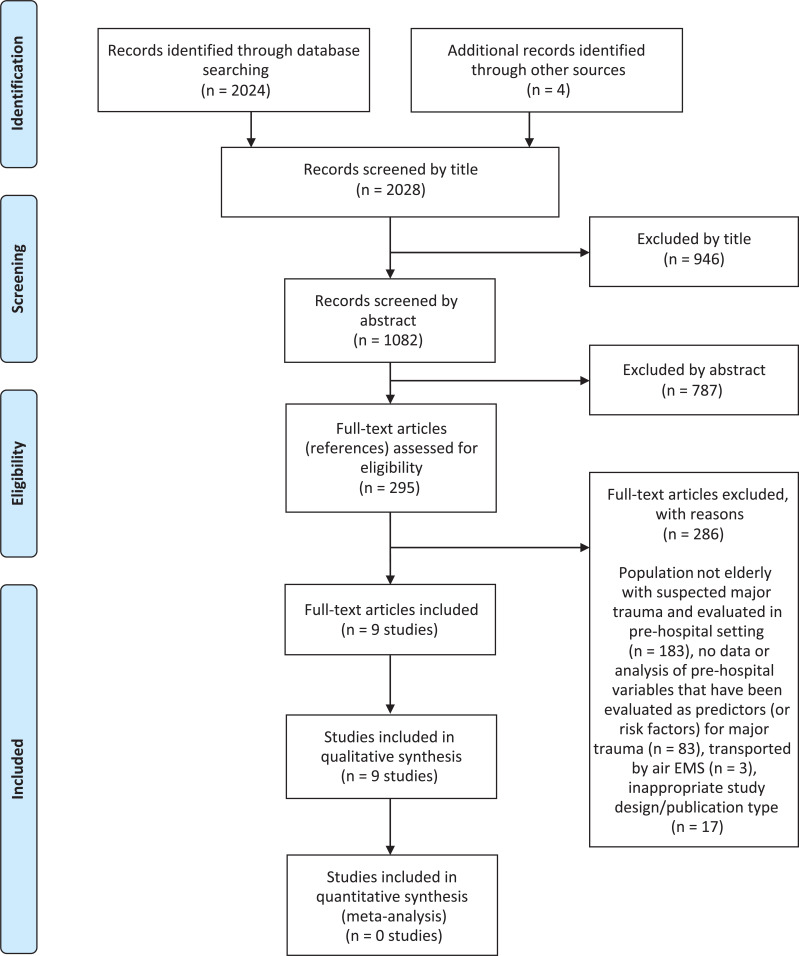
Figure 1. Study flow chart (adapted from Moher et al., 2009).

Figure 1 summarises the process of identifying and selecting relevant literature. Of the 2028 citations identified, 295 full-text articles were retrieved and fully assessed; nine studies (Benjamin et al., 2018; Brown et al., 2015; Caterino et al., 2011; Davidson et al., 2014; Newgard et al., 2012, 2014, 2019; Nishijima et al., 2017; Scheetz et al., 2007) met all the inclusion criteria. A full list of all excluded studies with reasons for exclusion is available on request.

### Study and patient characteristics

The design and patient characteristics of the nine included studies are summarised in Table 1. All studies were retrospective cohort studies undertaken in the United States and published between 2007 and 2019. While all studies included adult elderly trauma patients, the definition of elderly or older persons ranged between 55+ (Davidson et al., 2014; Newgard et al., 2012, 2014; Nishijima et al., 2017), 60+ (Benjamin et al., 2018), 65+ (Brown et al., 2015; Newgard et al., 2019; Scheetz et al., 2007) and 70+ (Caterino et al., 2011). The proportion of male patients ranged from 33% (Newgard et al., 2019) to 60% (Nishijima et al., 2017) (not reported in three studies) (Caterino et al., 2011; Davidson et al., 2014; Newgard et al., 2012). In studies that reported the cause of suspected major trauma in the elderly, the most common were blunt injuries caused by motor vehicle accidents (MVAs) and falls (Davidson et al., 2014; Newgard et al., 2014, 2019; Nishijima et al., 2017; Scheetz et al., 2007). Comorbidities and medication use (e.g. anticoagulants and/or antiplatelet therapy) were poorly reported. The percentage of severely injured patients ranged from 1.4% (Nishijima et al., 2017) to 32% (Brown et al., 2015) depending on definition, study design and type of participating hospital (not reported in three studies) (Caterino et al., 2011; Davidson et al., 2014; Newgard et al., 2012). All studies collected data on predictors through electronic trauma record systems and/or linked registries. Analysis and outcome for major trauma varied between studies and included measures such as an ISS ≥ 16 (four studies) (Davidson et al., 2014; Newgard et al., 2012, 2014; Scheetz et al., 2007), or composite injury outcomes (four studies) (Brown et al., 2015; Caterino et al., 2011; Newgard et al., 2019; Nishijima et al., 2017). A single study only looked at independent risk factors for early mortality after trauma (Benjamin et al., 2018). The association of individual predictors with major trauma was assessed through a classification and regression tree analysis (three studies) (Newgard et al., 2014, 2019; Scheetz et al., 2007), multi-variate logistic regression model (three studies) (Brown et al., 2015; Newgard et al., 2012, 2014), diagnostic accuracy metrics (five studies) (Brown et al., 2015; Caterino et al., 2011; Davidson et al., 2014; Nishijima et al., 2017; Scheetz et al., 2007) and descriptive statistics (one study) (Benjamin et al., 2018).

**Table 1.** Study and population characteristics.

Author, year	Design	Country	Sample size (elderly)	Elderly definition	Population	Mean/median age	Male	Mechanism of injury	Blunt trauma	Data collection [predictor ascertainment]	Data source	Predictors evaluated (n)	Statistical analysis	Outcome measurement
Scheetz et al., 2007	RCS	USA	7883	≥ 65	MVA victims	75	50%	RTA: 100%	NR	Prospectively collected crash site data. Retrospectively collected clinical data from EMS, police and hospital case records	NASS-CDS database	(26) age; air bag deployment; air bag type; alcohol involved; alcohol presence; ejection; entrapment; eyewear worn; fire; GCS; head restraint; height; injury severity (police-estimated); instrument panel damage; knee bolster damage; manner of collision; number of persons injured in the crash; occupant mobility; occupant’s seat position; other drug present; primary object contacted; restraint use; rollover; sex; weight; windshield glazing damaged	(a) diagnostic accuracy metrics; (b) classification and regression trees	ISS ≥ 16
Caterino et al., 2011	RCS	USA	15,708	≥ 70	Injuries requiring admission > 48 hours, interhospital transfer, or in hospital death	NR	NR	NR	NR	Retrospectively collected clinical data from EMS and hospital case records	Ohio Trauma Registry	(1) GCS	Diagnostic accuracy metrics	In-hospital mortality, traumatic brain injury, neurosurgical intervention or endotracheal intubation
Newgard et al., 2012	RCS	USA	NR	≥ 55	Unselected injuries	NR	NR	NR	NR	Retrospectively collected clinical data from EMS and hospital case records	WESTRN	(1) EMS provider judgement	Multi-variate logistic regression model	ISS ≥ 16
Newgard et al., 2014	RCS	USA	44,890	≥ 55	Unselected injuries	77	37%	Falls: 50%; RTA: 13%; Other/missing: NR	NR	Retrospectively collected clinical data from EMS and hospital case records	WESTRN	(5) GCS; respiratory rate; SBP; shock index; heart rate	(a) multi-variate logistic regression model; (b) classification and regression trees	ISS ≥ 16
Davidson et al., 2014	RCS	USA	12,435	≥ 55	MVA victims who did not meet step 1 or 2 criteria of US national field triage guidelines	NR	NR	RTA: 100%	NR	Prospectively collected crash site data. Retrospectively collected clinical data from EMS and hospital case records	NASS-CDS database	(8) intrusion occupant site; other intrusion; death in vehicle; steering wheel collapse; roof crush occupant site; roof crush other; ejection, entrapment	Diagnostic accuracy metrics	ISS ≥ 16
Brown et al., 2015	RCS	USA	438,828	> 65	Injuries meeting National Trauma Data Bank inclusion criteria	80	39%	NR	99%	Retrospectively collected clinical data from EMS and hospital case records	National Trauma Data Bank	(1) SBP	(a) multi-variate logistic regression model (b) diagnostic accuracy metrics	Trauma care need (composite of ISS > 15, ICU admission of 24 hours or greater, need for urgent surgery or death in the ED)
Nishijima et al., 2017	RCS	USA	1948	≥ 55	Head-injured patients who did not meet step 1 or 2 criteria of US national field triage guidelines	73	60%	Falls: 72%; RTA: 17%; Other: 11%	100%	Retrospectively collected clinical data from EMS and hospital case records	EMS electronic records. Hospital inpatient records	(1) Anticoagulants	Diagnostic accuracy metrics	Death or neurosurgical intervention during hospitalisation
Benjamin et al., 2018	RCS	USA	358,504	> 60	Stable blunt trauma injuries meeting National Trauma Data Bank inclusion criteria	NR	41%	NR	100%	Retrospectively collected clinical data from EMS and hospital case records	National Trauma Data Bank	(1) Comorbidity	Univariable association	Mortality
Newgard et al., 2019	RCS	USA	5021	≥ 65	Unselected injuries	82	33%	Falls: 83%; RTA: 8%; Other 9%	NR	Retrospectively collected clinical data from EMS, Medicare and hospital case records	EMS electronic records,state trauma, discharge and death registries	(6) GCS; SBP; respiratory rate; heart rate; comorbidities; anticoagulants	Classification and regression trees	ISS ≥ 16 or need for major non-orthopaedic surgical intervention

EMS = emergency medical services; GCS = Glasgow Coma Scale; ICU = intensive care unit; ISS = Injury Severity Score; MVA = motor vehicle accident; NASS-CDS = National Automotive Sampling System – Crashworthiness Data System; NR = not reported; RCS = retrospective cohort study; RTA = road traffic accident; SBP = systolic blood pressure; USA = United States of America; WESTRN = Western Emergency Services Translational Research Network.

### Risk of bias assessment and generalisability

**Figure 2:**
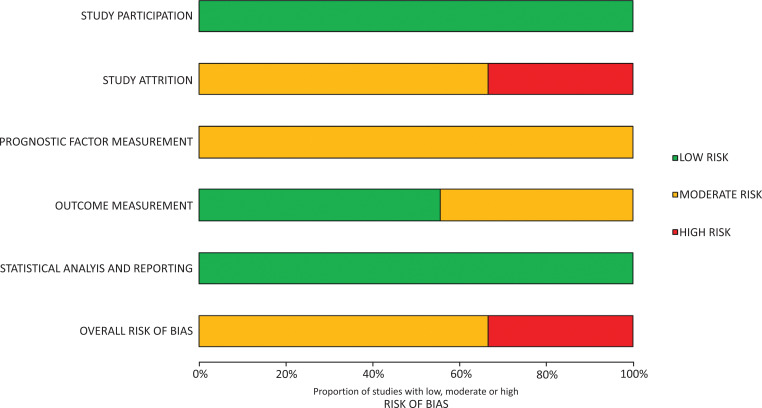
Figure 2. QUIPS assessment summary graph: review authors’ judgements.

The overall methodological quality of the nine included studies is summarised in Table 2 and Figure 2 (further details of review author judgements can be found in Supplementary 2). The methodological quality of the included studies, as assessed using the QUIPS tool, was variable. All studies were at moderate to high risk of bias. The main sources of systematic error were potential selection bias arising from missing pre-hospital data, and possible information bias from misclassification of predictor variables or outcomes secondary to retrospective abstraction of routine clinical records in trauma registry data.

**Table 2.** QUIPS quality assessment summary: review authors’ judgements.

Author, year	Risk of bias					
	Study participation	Study attrition	Prognostic factor measurement	Outcome measurement	Statistical analysis and reporting	Overall
Scheetz et al., 2007	LOW	MODERATE	MODERATE	LOW	LOW	MODERATE
Caterino et al., 2011	LOW	HIGH	MODERATE	MODERATE	LOW	HIGH
Newgard et al., 2012	LOW	MODERATE	MODERATE	MODERATE	LOW	MODERATE
Newgard et al., 2014	LOW	MODERATE	MODERATE	MODERATE	LOW	MODERATE
Davidson et al., 2014	LOW	MODERATE	MODERATE	LOW	LOW	MODERATE
Brown et al., 2015	LOW	MODERATE	MODERATE	LOW	LOW	MODERATE
Nishijima et al., 2017	LOW	HIGH	MODERATE	LOW	LOW	HIGH
Benjamin et al., 2018	LOW	HIGH	MODERATE	LOW	LOW	HIGH
Newgard et al., 2019	LOW	MODERATE	MODERATE	MODERATE	LOW	MODERATE

### Pre-hospital variables as prognostic factors or diagnostic tests for major trauma

A summary of the identified individual pre-hospital variables and their association with major trauma in elderly patients presenting to EMS following injury are provided in Table 3. Identified studies examined vital signs (four studies) (Brown et al., 2015; Caterino et al., 2011; Newgard et al., 2014, 2019), motor vehicle crash scene variables (two studies) (Davidson et al., 2014; Scheetz et al., 2007), EMS provider judgement (one study) (Newgard et al., 2012), anticoagulant use (two studies) (Newgard et al., 2019; Nishijima et al., 2017) or patient comorbidities (two studies) (Benjamin et al., 2018; Newgard et al., 2019).

**Table 3.** Relationship between identified pre-hospital variables and major trauma.

Author, year	Measure of association used	Outcome	Prognostic factor(s)	Results (95% CI, where reported)
Scheetz et al., 2007	Sensitivity and specificity	ISS ≥ 16	> 1 occupants injured	Sensitivity, 74.4% (68.0–80.1); Specificity, 45.6% (44.3–47.0)
			Occupant not independently mobile	Sensitivity, 93.8% (88.9–97.0); Specificity, 71.4% (70.2–72.5)
			Alcohol involved	Sensitivity, 14.0% (9.3–20.0); Specificity, 95.9% (95.3–96.3)
			Drug use	Sensitivity, 8.6% (4.6–14.2); Specificity, 98.5% (98.2–98.8)
			Restraints not used	Sensitivity, 35.3% (28.7–42.4); Specificity, 92.2% (91.6–92.8)
			Head-on collision	Sensitivity, 17.4% (12.5–23.1); Specificity, 97.9% (97.5–98.2)
	Significant variables in CART analysis	ISS ≥ 16	> 1 occupants injured Occupant not independently mobile Alcohol involved Drug use Restraints not used Head-on collision	Occupant not mobile, head-on collision, other injured occupants selected as significant prognostic factors
Caterino et al., 2011	Sensitivity and specificity	Mortality	GCS ≤ 14	Sensitivity, 59.2% (56.1–62.3); Specificity, 85.1% (84.6–85.7)
			GCS ≤ 13	Sensitivity, 50.7% (47.5–53.9); Specificity, 93.8% (93.4–94.2)
		Neurosurgical intervention	GCS ≤ 14	Sensitivity, 49.7% (42.6–56.9); Specificity, 82.8% (82.2–83.4)
			GCS ≤ 13	Sensitivity, 42.7% (35.7–49.9); Specificity, 91.5% (91.0–91.9)
		Intubation	GCS ≤ 14	Sensitivity, 66.3% (62.2–70.1); Specificity, 84.2% (83.6–84.8)
			GCS ≤ 13	Sensitivity, 57.5% (53.3–61.6); Specificity, 92.9% (92.5–93.3)
Newgard et al., 2012	Adjusted odds ratio	ISS ≥ 16	EMS provider judgement	Adjusted OR=1.23 (1.03–1.47)
Newgard et al., 2014	Univariable association	ISS ≥ 16	GCS score SBP Respiratory rate Shock index Heart rate	Significant non-linear relationships p < 0.001: GCS score – monotonically decreasing SBP, heart rate, shock index – u-shaped Respiratory rate – j-shaped
	Multi-variable association	ISS ≥ 16	GCS score SBP Respiratory rate Shock index Heart rate	Significant non-linear relationships, p < 0.001: GCS score, SBP, respiratory rate, shock index Non-significant relationship, p=0.48: Heart rate
	Importance of variables in CART analysis	ISS ≥ 16	GCS score SBP Respiratory rate Shock index Heart rate	Important variables and optimal cut-off point: GCS score ≤ 14 Respiratory rate < 10 or > 24 breaths per minute SBP < 110 or > 200 mmHg Shock index > 1.0 Heart rate ranked low in variable importance
Davidson et al., 2014	Sensitivity and specificity	ISS ≥ 16	Intrusion > 12 inches occupant site	Sensitivity, 47.8% (42.4–53.3); Specificity, 62.7% (59.2–66.3)
			Intrusion > 18 inches anywhere	Sensitivity, 10.9% (9.3–12.4); Specificity, 66.5% (63.2–69.9)
			Death in vehicle	Sensitivity, 28.5% (24.9–32.1); Specificity, 86.1% (84.6–87.6)
			Steering wheel collapse	Sensitivity, 0.8% (0.59–1.1); Specificity, 99.6% (99.4–99.7)
			Roof crush > 12 inches occupant site	Sensitivity, 5.1% (4.1–6.0); Specificity, 75.6% (72.5–78.8)
			Roof crush > 18 inches anywhere	Sensitivity, 1.8% (1.2–2.4); Specificity, 90.1% (97.6–98.8)
			Ejection	Sensitivity, 23.13% (19.1–27.2); Specificity, 63.7% (59.0–68.4)
			Entrapment	Sensitivity, 9.9% (8.5–11.4); Specificity, 69.4% (65.0–73.8)
Brown et al., 2015	Sensitivity and specificity	Composite outcome	SBP < 110 mmHg	Sensitivity, 13%; Specificity, 93%
		ISS ≥ 16, ICU admission > 24 hours, need for urgent surgery	SBP < 90 mmHg	Sensitivity, 5%; Specificity, 99%
Nishijima et al., 2017	Sensitivity and specificity	Composite outcome: in-hospital death or neurosurgery	Anticoagulation use	Sensitivity, 56% (35.3–74.5); Specificity, 71% (69.2–73.3)
Benjamin et al., 2018	Univariable association	Mortality	Comorbidities Cerebrovascular accident Congestive heart failure	Significant association between prognostic factor and outcome, p < 0.001
Newgard et al., 2019	Binary recursive partitioning	ISS ≥ 16 or major non-orthopaedic surgical intervention	Number of comorbidities Anticoagulant use GCS SBP Respiratory rate Heart rate	Identified as primary predictor variables: GCS ≤ 14 SBP < 110 or > 200 mmHg Respiratory rate < 10 or > 24 breaths/min Heart rate < 60 or > 110 beats/minute ≥ 2 comorbidities Not identified as a primary predictor variable: Anticoagulant use

CART = classification and regression trees; EMS = emergency medical services; GCS = Glasgow Coma Scale; ICU = intensive care unit; ISS = Injury Severity Score; SBP = systolic blood pressure.

Glasgow Coma Scale (GCS) score, systolic blood pressure (SBP), respiratory rate and shock index were identified as significant pre-hospital variables associated with major trauma in the elderly. Further examination of vital signs suggested optimal cut-off points of GCS score ≤ 14, respiratory rate < 10 or > 24 breaths per minute, SBP < 110 or > 200 mmHg and shock index > 1.0. Other significant pre-hospital variables comprised EMS provider judgement, comorbidities and certain crash scene variables (other occupants injured, occupant not independently mobile and head-on collision). Heart rate and anticoagulant use did not appear to be important predictors.

## Discussion

### Summary of results

In a systematic review of nine observational studies evaluating the association of pre-hospital variables with major trauma in elderly patients, we found that vital signs (GCS score, SBP, respiratory rate and shock index with specific elderly cut-off points), EMS provider judgement, comorbidities and certain crash scene variables (other occupants injured, occupant not independently mobile and head-on collision) were identified as significant pre-hospital variables associated with major trauma in the elderly. Heart rate and anticoagulant use did not appear to be important predictors. Included studies were at moderate or high risk of bias, and differences in study design, study populations and definition of major trauma make comparisons of the evidence difficult. Nevertheless, these findings may be used to inform development of future elderly major trauma triage tools or prediction models, and will inform EMS clinician triage decisions in the field.

### Interpretation of results

Major trauma is increasing in incidence in older adults and is recognised as a significant public health challenge in developed health systems (Kehoe et al., 2015). Recent systematic reviews have demonstrated suboptimal performance for existing major trauma triage tools and a paucity of elderly-specific tools (Fuller et al., 2021; van Rein et al., 2017, 2018). High levels of under-triage mean that initial treatment is more likely to be in non-MTC, and to be undertaken by less experienced doctors (Banerjee et al, 2017). Triage tools for injured patients have traditionally been developed through expert consensus, with more recent attempts to develop multi-variable prediction models (van Rein et al., 2019). In either case, this systematic review summarises the available empirical data on which to consider candidate triage variables.

Vital signs are a core element of existing triage tools and the observed interaction between increasing age and observed physiology with the probability of major trauma is therefore important (van Rein et al., 2017). Normal physiological ranges change during ageing and may be affected by comorbidities or medications, potentially resulting in sub-optimal cut-off points for physiological triage variables in the elderly. Technological advances, for example mobile applications, may allow the full relationship between age, vital sign values and risk of major trauma to be incorporated (van Rein et al., 2019). Alternatively, different vital sign cut-off points for elderly patients could be considered in traditional triage tools. Using thresholds of GCS ≤ 14 and SBP < 110 mmHg has been suggested (Brown et al., 2015; Caterino et al., 2011; Newgard et al., 2019), which would increase sensitivity, but potentially lead to unacceptable levels of over-triage.

Although MVAs are a relatively uncommon mechanism of injury in the elderly, the absolute numbers are increasing in ageing developed world populations (Azami-Aghdash et al., 2018). Interestingly, some crash scene variables included in the current US Field Triage Decision Scheme (intrusion, ejection) appeared to have sub-optimal performance in the elderly compared to younger adults (Sasser et al., 2009). Conversely, the presence of non-included variables (other injured occupants and victims not being independently mobile) were identified as possibly important pre-hospital variables to identify major trauma in this subgroup of elderly patients. This finding would benefit from future confirmatory studies.

The reported lack of association between anticoagulant use and presence of major trauma suggests that anticoagulant use may not influence the risk of developing major trauma (although it could influence injury severity if major trauma is sustained). Alternatively, this finding could be secondary to selection bias due to exclusion of patients without pre-injury medication information available and/or that the predictive utility of anticoagulation was reduced by including comorbidity information in multi-variable analyses. Furthermore, anticoagulation use was determined according to receipt of outpatient prescriptions, and it has been well established that adherence to these drug regimens can be as low as 50% (Abdou et al., 2016). Finally, newer direct oral anticoagulants have demonstrated an improved safety profile compared to older vitamin K antagonists (Gupta et al., 2019).

The predictive value of age itself in major trauma has been investigated in two recent systematic reviews. Hashmi et al. (2014) demonstrated that increasing age is associated with higher mortality following injury, while Brown et al. (2020) reported increasing rates of triage to non-MTCs in older injured patients. However, advancing age is a non-specific marker of poor outcome across all disease areas, and mortality as an endpoint may not reflect the anatomical burden of injury or the potential for benefit from MTC care; although it is possible that older adults may not receive aggressive care secondary to perceived poor outcomes. Interestingly, a recent study investigating injured patients conveyed by EMS in the Netherlands suggested a bell-shaped pattern with the probability of major trauma peaking at 40–50 years and declining in younger and older age groups (van Rein et al., 2018).

The pre-hospital identification of major trauma triage in the elderly is challenging due to the confounding effects of frailty, aging, comorbidities, polypharmacy and concurrent acute medical conditions. Mechanism and pattern of injury also differ from younger trauma patients, with ground-level falls and blunt head injury pre-dominating (Kehoe et al., 2015). It is therefore likely that novel pre-hospital variables are required to improve triage accuracy. Potential prognostic factors might be identified through study of false positive and negative cases, or through qualitative research with receiving hospital doctors. Ultimately, it may be necessary to acknowledge that it is not possible to achieve American College of Surgeons Committee on Trauma (ACS-COT) suggested targets for under-triage (< 5%) and over-triage (< 25–35%) in the elderly (American College of Surgeons, 2006), and to focus efforts on robust secondary transfer protocols.

Major trauma is traditionally defined anatomically using an ISS threshold of ≥ 16 (Palmer, 2007). However, as the ISS does not fully account for injury acuity, prognosis or futility, it has limitations as a measure for identifying seriously injured patients who could benefit from MTC care. Mortality is also a problematic endpoint for evaluating major trauma triage. The premise that older adults will have the same benefit from MTC care as younger adults is unproven, with the US Costs and Outcomes of Trauma study failing to identify a significant improvement in mortality for the elderly population (Mackenzie et al., 2007). For older patients with un-survivable injuries, or very severe comorbidities, outcomes may be fixed regardless of specialist care, making bypass away from local non-specialist hospitals futile. For other injured older adults, particularly in the context of advanced frailty, the probability of improved outcome may be low compared to the burden of treatment, and advanced MTC care might not be in a patient’s best interests. Patients and families may also prefer care closer to home in a local hospital and be willing to ‘trade’ better overall outcome to achieve this. Reference standards including resource use and frailty scores (Rockwood et al., 2005) might be used in future studies to give a better evaluation of pre-hospital triage variable performance in the elderly.

This review included studies from a range of established trauma systems in the United States, and the findings should be largely generalisable to other similar health services in the developed world. However, several studies included highly selected populations (stable patients following MVAs, stable head injured patients) and/or examined narrow trauma registry populations, rather than all injured patients presenting to EMS, potentially reducing generalisability to the wider population.

### Comparison to the existing literature

This is the first systematic review examining individual pre-hospital variables as prognostic factors or diagnostic tests. However, recent systematic reviews have evaluated overall triage tool performance in elderly patients. Fuller et al. (2021) included 15 observational studies investigating both general triage tools and elderly-specific instruments (Fuller et al., 2021). The diagnostic performance of the triage protocols was highly variable, with differences in study design, study populations and reference standard making comparisons difficult. Boulton et al. (2021) reported similar findings from their systematic review of 11 included studies on elderly-specific pre-hospital trauma triage tools and concluded there was uncertainty over the optimal elderly triage tool. Triage accuracy fell below ACS-COT-suggested triage targets for under-triage (< 5%) and over-triage (< 25–35%) in most included studies in each review. The studied elderly tools included the same prognostic factors identified in the current review (comorbidities, anticoagulant use and alternative physiology thresholds), highlighting the importance of future investigation of novel pre-hospital variables to improve future triage tools. Overall, modifying the Field Triage Decision Scheme with elderly-specific vital sign thresholds and variables was shown to increase sensitivity for detecting injured patients with ISS ≥ 16 from 78.6% to 86.3%, at a cost of reduced specificity (75.5% to 60.7%) (Newgard et al., 2014; Sasser et al., 2012). Whether this trade-off is desirable will depend on the valuation of false positives/negatives, the costs and consequences of under-/over-triage, the incidence of injured patients presenting to EMS and the prevalence of major trauma.

### Strengths and limitations

This systematic review has a number of strengths. It is the first study to identify individual risk factors and predictors that are likely to increase the risk of major trauma in the elderly, and was conducted with robust methodology in accordance with established guidelines for undertaking prognostic factor systematic reviews (Riley et al., 2019). However, there are several potential weaknesses. We did not perform hand-searching (i.e. manual page-by-page examination of the entire contents) of journals or conference proceedings, and did not include regional bibliographic databases, although the yield of such searches is generally low (Lefebvre et al., 2020). Decisions on study relevance, information gathering and validity were unblinded and could potentially have been influenced by pre-formed opinions. However, masking is resource intensive with uncertain benefits (Morissette et al., 2011). Finally, we included studies enrolling participants < 60 years of age if the ratio was > 75%, which may reduce the applicability of results.

## Conclusions

Existing studies examining the association of individual pre-hospital variables with major trauma in elderly patients are at moderate or high risk of bias and may have limited generalisability to injured patients presenting to EMS. Vital signs (GCS score ≤ 14, SBP < 110 mmHg, respiratory rate < 10 or > 24 breaths per minute and shock index > 1), EMS provider judgement, comorbidities and certain crash scene variables (other occupants injured, occupant not independently mobile and head-on collision) were identified as significant pre-hospital variables associated with major trauma in the elderly. Heart rate and anticoagulant use did not appear to be important predictors. These findings will guide selection of candidate predictors when developing future elderly major trauma triage tools or prognostic models. Furthermore, the association between higher vital sign thresholds and major trauma in the elderly could inform the assessment of injured older adults by EMS clinicians in the field. Future work to improve pre-hospital trauma triage tools in the elderly could focus on a more valid reference standard reflecting the need for MTC care rather than injury severity; including elderly-specific physiology thresholds; and further evaluation of novel elderly relevant triage tool variables and thresholds.

## Author contributions

AP co-ordinated the study. GF and AP were responsible for conception, design and obtaining funding for the study. HBW developed the search strategy, undertook searches and organised retrieval of papers. AP, ME, LS, HC, CH and GF were responsible for the acquisition, analysis and interpretation of data. GF helped interpret and provided a methodological, policy and clinical perspective on the data. GF and AP were responsible for the drafting of this article, although all authors provided comments on the drafts and read and approved the final version. GF acts as the guarantor for this article.

## Conflict of interest

None declared.

## Ethics

Not required.

## Funding

This study was funded by the United Kingdom National Institute for Health Research Health Technology Assessment Programme (project number 17/16/04). The views expressed in this report are those of the authors and not necessarily those of the NIHR HTA Programme. Any errors are the responsibility of the authors. The funders had no role in the study design; in the collection, analysis and interpretation of data; in the writing of the manuscript; or in the decision to submit the manuscript for publication.
